# An effective approach to tackling complex health policy challenges. Using a clinical microsystems approach and rethinking codesign

**DOI:** 10.3389/fpubh.2024.1405034

**Published:** 2024-10-23

**Authors:** M. Duggan, J. A. Dunbar, M. A. Morgan, K. P. Mc Namara, M. P. de Courten, R. V. Calder

**Affiliations:** ^1^Australian Health Policy Collaboration, Victoria University, Melbourne, VIC, Australia; ^2^Deakin Rural Health, Warrnambool, School of Medicine, Deakin University, VIC, Australia; ^3^Faculty of Health Sciences and Medicine, Bond University, Gold Coast, QLD, Australia

**Keywords:** health policy, clinical microsystem, mental health, physical health, integrated healthcare, healthcare disparities, systems approach, lived experience

## Abstract

That people with serious mental illness have poor physical health and face a significant life expectancy gap compared with the general population is well known. Despite considerable policy focus in some countries, the gap in life expectancy remains. Tackling complex and persistent health problems such as this requires a systems-based approach, recognising the complexity of interacting components and their effects on the problem and on each other and applying collaborative analysis, design and implementation by those with knowledge of and expertise in the problem and the context. This paper describes the methods used to develop the Australian Being Equally Well National Policy Roadmap for better physical health care and longer lives for people with severe mental illness. Whilst recognising that high rates of physical health comorbidities are caused by many factors including lifestyle, access to high-quality healthcare and medication side effects, the work was focused on what could be done within Australian primary care to improve the physical health of this cohort. A Clinical Microsystem Approach was applied to synthesise clinical evidence with professional and lived experience, and an innovative policy development process was established, creating trust across all system levels. Participants with different kinds of knowledge and experience worked in discrete groups according to their professional or expert role whilst also being supported to participate in an intensive cross-collaboration. The potential value of this methodology for tackling other complex problems in health policy is discussed.

## 1 Introduction

People with severe mental illnesses have poorer physical health and reduced life expectancy compared to their peers (1). This is an international problem that has drawn policy attention in several countries, with little or no reduction in the gap in life expectancy or the burden of chronic disease.

International experts have defined key overarching risk factors across multiple social ecological levels and proposed a range of actional solutions for this persistent health problem (2). This paper has a more limited purpose. It describes the methods used to develop implementable ways of addressing the vital, if narrower, challenge of improving the quality of health care to people with severe mental illnesses. This is one of the six essential elements defined in the National Consensus Statement, developed by the Australian National Mental Health Commission for improving the physical health and well-being of people with mental illness (3).

Australia has identified closing this gap as a national priority. The 5th National Mental Health and Suicide Prevention Plan acknowledges that poor outcomes for people with serious mental illness are often ‘service driven with unnecessary barriers between health services and unclear delineation between professional roles hindering a consumer’s ability to get what they need’ (4).

Despite these policy efforts, the gap in life expectancy between people with serious mental illness and the general population is widening globally (5). The separation and disconnection between mental health and physical health care service provision and utilisation is recognised as a principal contribution to this (6).

In Australia, people living with serious mental illness die 10–15 years earlier than their peers (7). As in the general population, cardiovascular diseases and cancer are the two major causes of these deaths. Many of these early deaths are preventable.

The absence of significant impact from current policy is likely due to policies that do not create meaningful change in clinical care. Clinical Microsystems are where clinical care is provided and accessed by people with health care needs. It is here that quality and safety are built and health care outcomes improved. Policy approaches that rely on establishing incentives and measuring outcomes do not, of themselves, achieve fundamental change in clinical care and health outcomes (8). This is the essence of the Clinical Microsystem approach taken for this approach.

The Being Equally Well policy development project set out to achieve a consensus between people with lived experience, clinicians, administrators, researchers and policy experts on what could be done within Australia’s current health system to achieve effective improvements in physical health care for individuals with severe mental illnesses. The methodology for the project was based on a systems approach to health care design (9, 10) that centred on clinical microsystems to address gaps and barriers at the frontline of care for consumers and clinicians (9, 11–13).

The strengths and limitations of the methodology chosen to facilitate the project’s objective have lessons for those interested in avoiding policy failure concerning complex and resistant health challenges.

### 1.1 The policy problem: the complexity of health conditions and fragmentation of health services

One explanation suggested for policy failure is that policy development and implementation processes may be simplistic in the approach to so-called ‘wicked problems’. Wicked problems are complex, have multiple causes and are resistant to amelioration (14).

The persistent inequities in health and life expectancy between people with serious mental illnesses and their peers have many of the characteristics of a ‘wicked problem’—that is, a social system problem that is difficult to address, is poorly formulated, with multiple clients and decision makers with conflicting values that can contribute to greater complexity and poor outcomes (15). Successful policy implementation in this area, as in the whole field of mental health, requires changes in a highly complex service landscape involving parallel and distinct systems of health care—physical and mental health services—and several different levels of healthcare within each, with a wide range of clinical and non-clinical stakeholders responding to individuals with multi-factorial health needs.

Other explanations for policy failure focus on apparent inadequacies in traditional methods of health policy development, which have tended to follow a linear pathway from the formulation of policy intentions and aims through to the development of means and instruments to achieve those intentions (16). These processes may place more attention on policy design than on mechanisms for achieving the goal (17). Another explanation is that traditional approaches to policy development frequently set out the components of new policy without accompanying implementation plans or support for those who are expected to achieve implementation (17).

Australia’s Fifth National Mental Health and Suicide Prevention Plan elevated the physical health needs of people living with mental illness as a priority area (4). The Plan is the agreed outcome of a consultative process involving national, state and territory health agencies as well as health system and consumer/patient stakeholders. It sets out a series of actions for Australia’s federal, state and territory governments and services which are presented as shared intentions that include process or output measures relevant to the period of the national plan. In particular, the National Mental Health Plan proposes a range of areas for change without specifying how the structural and behavioural barriers, which stand in the way of the intended goals, will be modified by support for the proposed new ways of working. This fits, at least in part, a criticism of simplistic policymaking, which asserts that top-down exhortations to change are not sufficient by themselves (18).

### 1.2 Disjointed health services are the barriers

The health system components involved in responding to the physical health needs of people with serious mental illness are particularly complex and disjointed. They include multi-disciplinary specialist clinicians, non-specialist health professionals and lived experience workers and services in primary, secondary and tertiary health settings. There are historical, cultural, financial and policy-driven structural gaps between acute and continuing mental health care and general physical health care. Specialist mental health services, for example, may not consider that they have a role in physical health and may have limited capacity to do so (19). Other health services that are accessed by people with mental health problems may not generally be recognised as components of the mental health system (20).

General practise is well-placed to undertake preventive care and management of chronic health conditions that could improve health outcomes for this group (21). Fragmented care is a common experience with responsibility for physical health needs often falling into the cracks between specialist mental health teams and general practise (22). There is no historical practise of information sharing between mental health services and general practise in Australia, and where endeavours are made to bridge that gap, the lack of infrastructure and policy frameworks makes these hard to sustain in the longer term (19). In the Australian context, the maldistribution of psychiatrists with few working within remote, rural and deprived inner city areas adds to the barriers (23). Improving health outcomes requires systematic support for both mental and physical health care needs with clarity about who does what, for whom, when and how.

The United Kingdom introduction of the Quality and Outcomes Framework (QOF) for cardiovascular disease (CVD) management in 2004 resulted in a large improvement in CVD outcomes. The association between the quality of primary care and coronary heart disease outcomes was strongest in practises serving deprived populations. High-quality primary care appears to reduce inequalities in health outcomes, particularly for cardiovascular disease and diabetes (24, 25).

It was always likely that QOF would reduce cardiovascular and other chronic disease events. What is remarkable is the reduction in health disparities. One conspicuous conclusion is that any policy to reduce cardiovascular or other chronic disease events needs effective programmes for managing individuals at high risk (26).

## 2 Methods: a systems approach to redesigning health services

The Being Equally Well Project aimed to find ways of enhancing disease management approaches in Australian health care. The methodology that was developed applied systems thinking and theories of co-production and collaboration in health policy development to create an alternative approach to top-down, linear design and to systematically consider the four major contributors to health policy failure (17), namely:

1. overly optimistic expectations;
2. implementation in dispersed governance;
3. inadequate collaborative policymaking; and
4. the vagaries of the political cycle.

To enable the communities of knowledge to develop and inform each other through the project, the project methodology hybridised three approaches. These comprised:

- the Clinical Microsystem Approach to health system improvements,
- consumer and carer experience and expertise in health care provision, access and gaps, and
- thematic analysis to ensure that the recording and analysis of the knowledge exchange between different groups of participants was comprehensive, transparent and able to be interrogated by all parties in real-time.

The systems approach to redesigning health services recognises the multiplicity of elements interacting to impact the outcome of interest and can be structured around the three levels of the health system (micro, meso, and macro). The focus of this approach was to address health delivery challenges at the clinical microsystem level to improve both patient and service outcomes.

Clinical microsystems are the small, functional frontline units that provide most healthcare to most people. They are the essential building blocks of larger organisations and of the health system. They are the place where patients, families and care teams meet.

To implement this method, the project was undertaken by six working groups comprising 55 participants. Three groups brought together people from each level of the health system:

- Microsystem: the teams at the frontlines of care where patients and their families meet the health system. Clinical Microsystems are where clinical care is provided and accessed by people with health care needs. It is here that health care outcomes are improved. These teams include general practise, acute and community mental health teams, pharmacy and allied health professionals.
- Mesosystem: primary healthcare organisations (Primary Health Networks, PHNs); Local Health Networks/Districts (LHN/Ds) that provide public hospital services across Australia and commonly include more than one public hospital in a geographical catchment.
- Macrosystem: federal, state and territory governments; National Mental Health Commission, Australian Health Ministers Advisory Council, private health insurance providers.

The roles of the macrosystem and mesosystem are to support the microsystem. The respective three working groups considered what was required to support microsystems to improve health outcomes.

A fourth expert group was asked how to use the methodology of quality improvement in healthcare and measurement for systematic and sustainable improvements. Although frontline staff may have the knowledge about what needs to change, sometimes they require support to put it into practise.

Consumers and carers, experts by experience, comprised the fifth and central working group. Even in the most traditional model of medical practise, outcomes are co-produced. The degree to which patients and professionals each hold agency for these co-produced outcomes varies widely but the observation that health outcomes depend on both parties seems self-evident. This concept has profound implications for improving healthcare quality, safety and value (27).

A sixth group, the System Integration Group, comprised the Project Director, the AHPC Research and Clinical Adviser, the national director of Equally Well Australia (28), the chairs and rapporteurs of each working group and the Thematic Analyst. Its role was to coordinate and integrate group outputs, analysis and reporting.

The initial task for all working groups was to separately identify the barriers and gaps each considered contributed to poor health outcomes. The outputs from these discussions were immediately shared through the System Integration Group. In the next phase, the expert consumer and carer group developed measures of success, providing outcome aspirations and indicators for five domains of health care that the group established as being central to improvement: improved physical health; management of medication impact; relationships with health professionals; system integration, support/equity of access and care quality; peer support (19). These were shared with and informed consideration by the clinical system expert groups. These groups considered how to address the identified barriers and gaps in light of the measures of success. Practically speaking, these discussions promoted a systems thinking approach to a shared understanding of how relevant micro-, meso- and macro-level factors, along with defined multilevel innovations, could interact and combine to shift the overall system dynamic towards supporting measurably improved access to and uptake of healthcare that will be effective in improving health outcomes.

**Figure 1:**
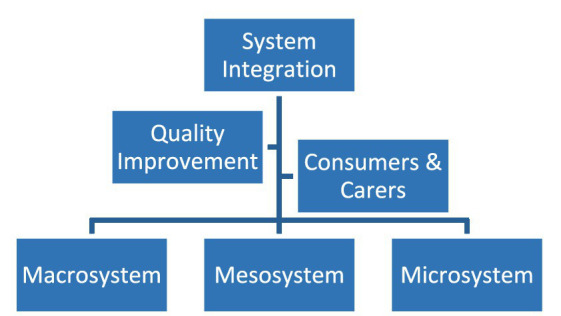
Being Equally Well: project working groups.

Figure 1 presents the organisational chart of the project expert working groups (19).

### 2.1 Designing for transparency and trust

The project process involved collaboration between multiple knowledge user communities, including consumers, carers, researchers, policymakers, practitioners and managers. This contrasts strongly with collaborative approaches which segment participants into two homogenous communities: knowledge producers and knowledge users (29). It also deployed a variant of current models of codesign which is increasingly promoted as best practise but for which there is limited empirical evidence of efficacy (30). Consumers raised concerns at the beginning of the process that working as a distinct, expert group, separately from the professionals, would marginalise their views. The literature identifies concerns about power, politics and perceptions as ‘non-trivial’ issues that require careful navigation and negotiation, particularly in relation to the differing perspectives that stakeholders may have about (*a*) the value and relevance of research and (*b*) respecting and understanding the contexts of the interpretation and application of research findings (31).

In responding to these concerns, and to emphasise equivalence between groups, the process was designed to support transparency and the growth of trust between the groups and participants. The strategies adopted for this included the appointment of a consumer co-chair for the project and the formal establishment of an iterative process which emphasised the views of consumers and carers at every meeting, and transparent feedback and summation at each stage. This included a joint meeting mid-project between the clinical working group chairpersons and all members of the consumers and carers working group in which the measures of success developed by the consumers and carers were vigorously and respectfully discussed. This meeting reinforced for all participants that the success of this project would emerge through the clinical groups listening to and understanding the experience, needs and aspirations of consumers and through collaborative design proposed healthcare service improvements.

The process enabled the emergence of mutual respect and a deep understanding of each other’s roles, contexts and contributions. Engagement in the work deepened as participants began to identify ‘what was in it’ for them (32), recognised as vital to successful collaboration.

The observed growth of mutual understanding and the emergence of consensus across the groups highlights that trust is indispensable for health systems to progress (33, 34). Trust improves collaborative decision-making for effective and sustainable partnerships (35, 36).

### 2.2 Thematic analysis

The complex structure of this project, involving five separate expert working groups undertaking four rounds of discussions over 6 months, reinforced the need for accurate transmission of information across groups. Thematic Analysis was the methodology chosen to enrich minute-taking and information sharing. It allowed real-time feedback to all groups about emerging themes in other groups.

Thematic analysis (TA) is a method applied to qualitative data, usually in recorded transcripts. Thematic analysis illustrates which themes are important. The result of a thematic analysis should be ‘to highlight the most salient constellations of meanings present in the dataset’ (37). TA established a defensible, rigorous and above all transparent process to identify the overt and latent themes emerging across all meetings and their relative importance for each group.

Issues raised were coded and clustered into major and minor themes by individual groups and across all the working groups. Codes were identified in an online workshop involving participants from each working group and analysed using Dedoose software that enables qualitative and mixed methods data analysis. Themes were weighted according to the frequency with which they were mentioned. The depth and richness of this analysis and the power of the graphical content and visualisations of the material enabled each of the working groups to see and understand the differing priorities of the other groups at the outset of the project. These priorities converged in time as the information-sharing process deepened.

Thematic analysis made the debates within each working group, including the tensions and obstacles, transparent. It assisted the development of openness and trust between the groups and the identification of sufficient common ground on which to build the final recommendations agreed to by all stakeholders as necessary, justified by the available evidence and responsive to the needs and demands of consumers.

## 3 Outcome: integrating evidence and experience

The Being Equally Well Project was a collaborative endeavour of mental and physical health professionals, service providers, advocates, consumers and carers, advocates, and policy experts. It was led by the Australian Health Policy Collaboration (AHPC), a national network of chronic disease and population health experts and consumers supported by the Mitchell Institute, a health and education policy think tank at Victoria University in Melbourne together with Equally Well (28)a network of over 90 organisations working together to make the physical health of people with mental illness a priority. Over a 6 months period of working together, participants achieved consensus on practical, evidence-based improvements that would reduce fragmentation of care and improve health outcomes and could be implemented nationally.

### 3.1 Setting shared and realistic expectations

The central question posed by the project was: ‘*What needs to change at the front lines of clinical care and how can the changes be supported?*’ The question acknowledged that current policies had not systematically improved the interface between clinician and patient nor achieved measurable improvements in health outcomes for people living with serious mental illness.

The focus on sustained, practical changes at the clinician-consumer interface set an appropriate ambition for the project. The question was understandable to both front-line clinicians and consumers. Primary care was recognised as the health service level responsible for identifying and managing the risk factors for the most prevalent causes of premature mortality, particularly cardiovascular disease and cancer.

The agreed aim of the project was to develop evidence-based and experience-informed, feasible and affordable proposals for policy initiatives for governments and health service providers that would remove or reduce barriers and establish effective care pathways to better health outcomes for people living with serious mental health conditions.

### 3.2 Collaborative policymaking

Having good evidence for potential system improvements was, and is, important. Clinical, administrative and consumer participants in this project all needed to have confidence in the identified solutions. This was facilitated by establishing parity between, and bringing together, equivalent kinds of knowledge (38):

- knowledge derived from research
- knowledge derived from audits and routinely collected data
- knowledge derived from the experience of patients, service users and professionals, also called lived experience.

The project design reflected the understanding that those working at the front lines of care ultimately determine the translation of policy into improved practise. By their actions and advocacy, they can influence the opinions, experiences and outcomes of patients and communities (39). This is the ‘bottom-up’ school of thought on policy implementation. Engagement of the ‘street level bureaucrat’ is vital in implementing policy (17). The front-line actor has ‘discretionary power (that) can prove instrumental in determining the success of a policy’ (40).

**Figure 2:**
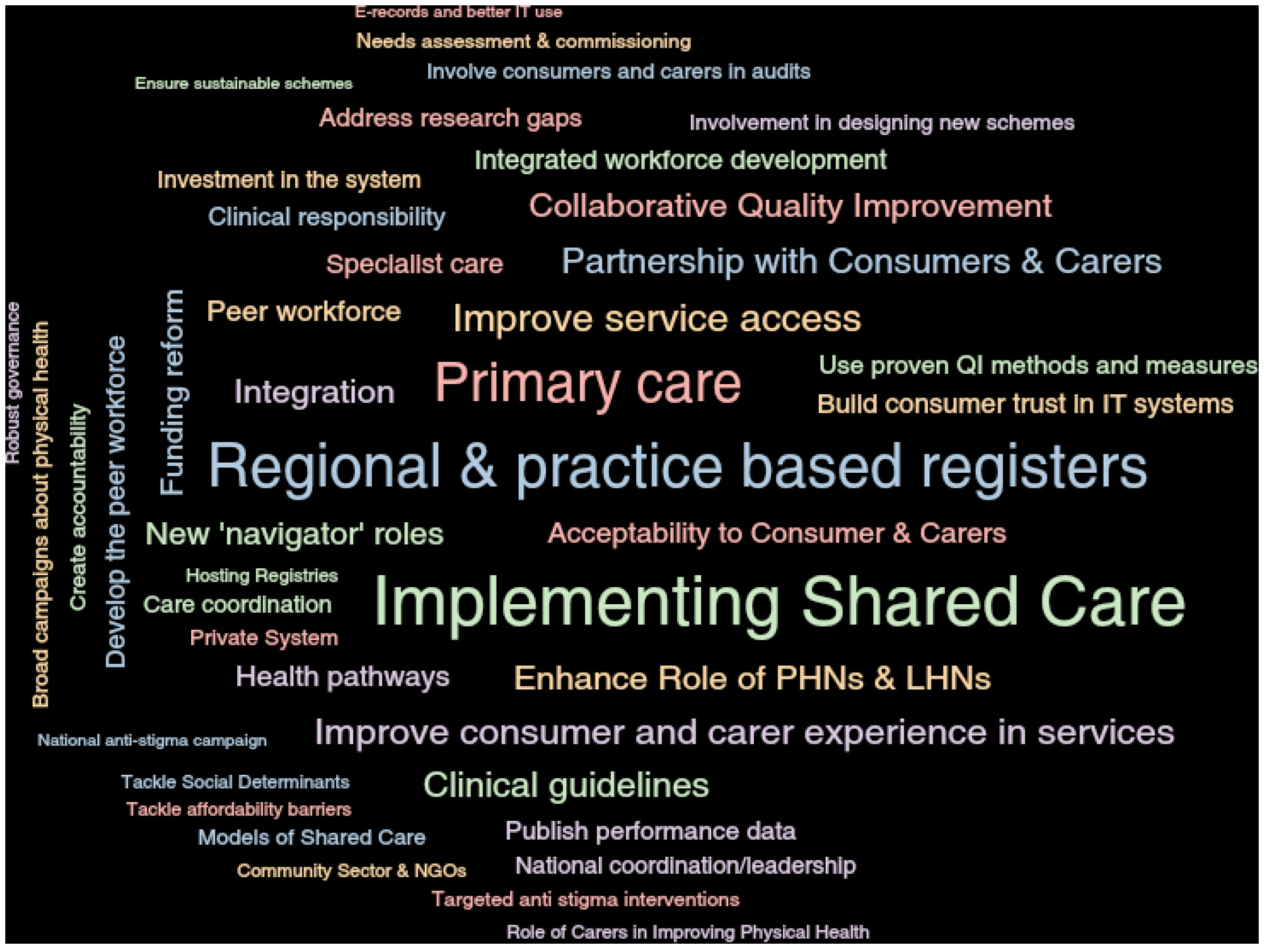
Word cloud of Roadmap priorities.

The priorities derived through thematic analysis at the summation of the process to build the final recommendations (Roadmap) are illustrated in Figure 2.

### 3.3 Implementation within dispersed governance

The Roadmap proposes that the barriers to systemic improvement that flow from dispersed governance and service provision should be offset by a system-wide quality improvement framework for performance measurement (6).

At the general practise level, data on key risk factors for this patient group would be returned via Primary Care Collaboratives to a National Mental Health Clinical Quality Register and by mental health services to state and national clinical quality registers. The Roadmap proposes that the Australian Commission for Safety and Quality in Health Care (21), a national central agency relevant to all levels and elements of Australian health services, provide the central data repository and clinical registry. This would aggregate data and provide both a national public annual report and performance reports back to individual clinical units allowing comparison with peer groups.

**Figure 3:**
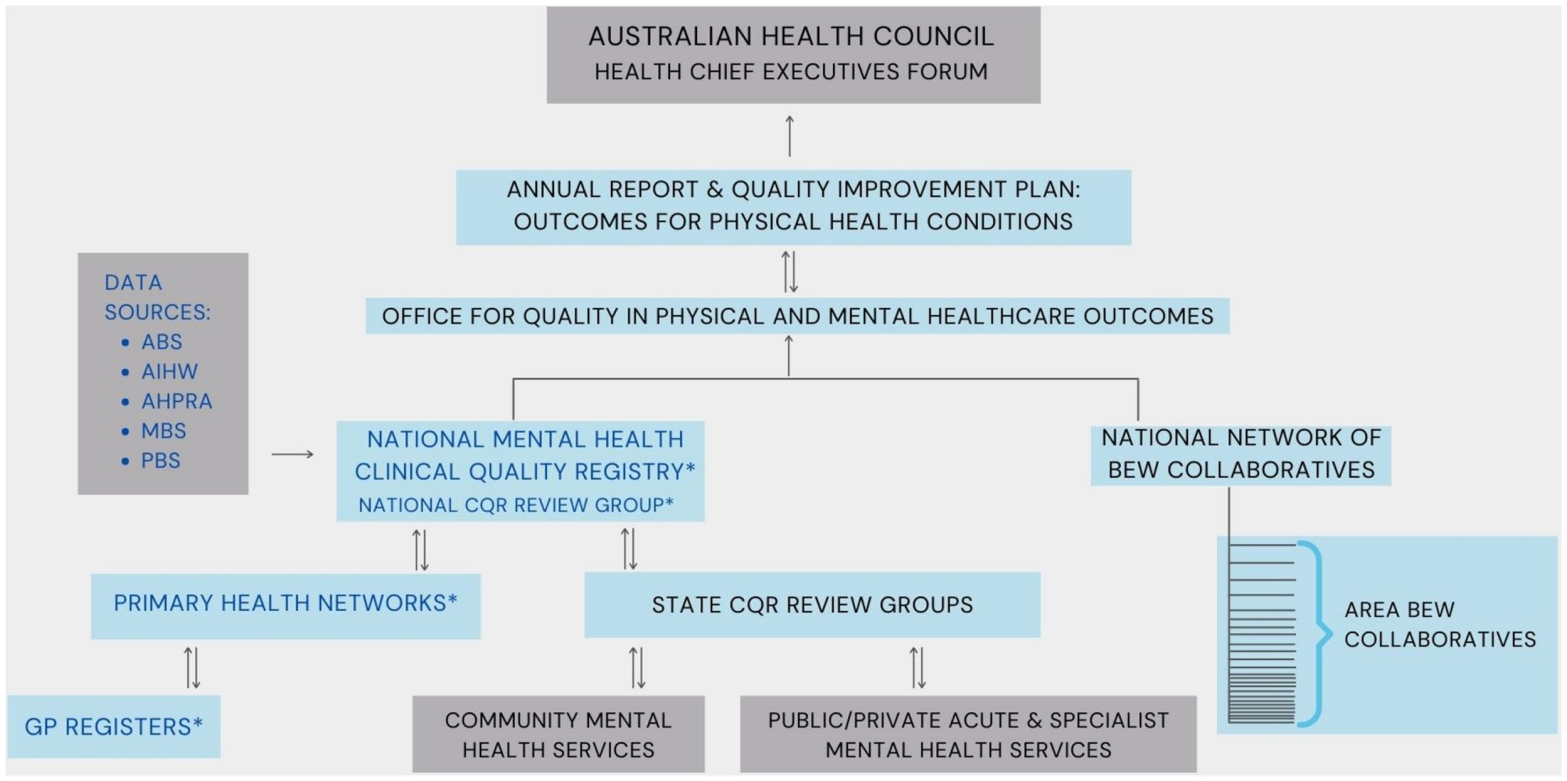
System diagram of the data flow to improve physical health outcomes among people with severe mental illness (19). Dark grey: Existing organisations/Components; light blue: Proposed organisations/Components; *Sources for local and national data. ABS, Australian Bureau of Statistics; AIHW, Australian Institute of Health & Welfare; AHPRA, Australian Health Practitioners Regulation Agency; BEW, Being Equally Well; CQR, Clinical Quality Registry; MBS, Medical Benefits Scheme; PBS, Pharmaceutical Benefits Scheme; PHNs, Primary Health Networks.

Figure 3 presents a diagram of the proposed system.

### 3.4 System improvements: Roadmap recommendations

Policies that require implementation at the frontline of care, between individuals and their immediate healthcare providers, must be designed with and for those at the frontline. The Roadmap recommendations describe precisely how workforce roles, financial incentives and technologies would need to be reshaped to achieve the policy goal. They also set out a process for regular monitoring, review and reporting on progress towards improved health outcomes and life expectancy.

The Being Equally Well Roadmap recommendations (6) include in summary:

- development and adoption of shared and integrated care guidelines between primary, secondary and tertiary levels of care;
- establishment of systems within primary care services for registration and periodic recall of individuals for risk factor identification and management;
- provision of interventions including medication management to address the cardiometabolic risks of antipsychotic and similar treatments;
- an additional workforce role of clinical care coordinators to support shared care provision between general practise, other primary health care including pharmacy and specialist mental health services and ensure that all consumers have a general practise home; and
- establishment of quality improvement collaboratives within primary health network catchment areas to facilitate peer engagement in quality improvement review, evidence and implementation. This approach was previously successful in improving general practise care of heart disease, diabetes, chronic obstructive pulmonary disease and other chronic health conditions (41) and demonstrated that general practise-based register and recall systems with reporting to and feedback from the collaboratives were effective in managing the health risks for patients with these conditions.

Details of the care coordination model, which was a major recommendation from the process, together with proposals for monitoring and evaluation of the process once implemented and the likely costs and other benefits are addressed within the Roadmap itself, particularly in the technical papers, and in the special supplement published by the Medical Journal of Australia in 2022 (42).

These changes need to be accompanied by financial incentives commensurate with the workload for primary care. The removal of financial barriers to medication access for consumers is essential. There is also a need for sustained support for quality improvement including the development of primary care collaboratives to address the physical health of people with severe mental illness at the front-line and a mechanism for scrutinising and reporting on progress with policy implementation to hold the system accountable at the national level.

## 4 Conclusion: an idea whose time has come?

In the 1990s, Kingdon said that, for an idea to flourish as a policy, it had to be ‘an idea whose time has come’ (43). For a patient or consumer group whose healthcare needs are commonly met by a separate and discontinuous health service system, recognition of the urgency of change will only come when there is consistent leadership and advocacy by healthcare professionals and advocates to achieve engagement by policymakers (44). This project set out to provide a strong consensus basis and framework for this.

To guide implementation leadership and pathways for the Being Equally Well recommendations, the AHPC and Equally Well collaboration undertook a year-long strategy to develop implementation advice and resources with a wider network of health professionals and services, together with the consumer and carer project working group. More than 200 experts from across the health sector participated in roundtables and workshops that developed a suite of detailed proposals for actions to facilitate the implementation of the Being Equally Well Roadmap recommendations. These were presented to policy-makers in national agencies through meetings and correspondence and by a submission, co-authored by the System Integration working group on behalf of the project, to the Australian Government 2024–25 pre-Budget process.

To date, developments towards implementation have included:

- A commitment in the Australian Government national budget in May 2024 of $AUD80 million in funding that addresses key recommendations for care coordination and support and a peer workforce for people with complex mental health needs. The budget initiative comprises: (i) mental health nurses and other allied health professional support in general practise to provide care coordination and support to patients with complex needs; (ii) a national peer workforce association to develop and professionalise the lived experience workforce. Additional initiatives to diversify the psychology workforce including the potential for psychology assistants in the mental health workforce; and provision of services for more than 18,000 people with severe mental illness who need psychosocial support also implement key elements of the project recommendations (45).
- Publication by the Medical Journal of Australia of a guest editorial and sponsored supplement, Being Equally Well: Ending the neglect of physical health for people with serious mental illness, summarising recommended system improvements and evidence relevant to improved primary care treatment and physical health care and outcomes (42, 46).
- A Targeted Call for Research by the Australian National Health and Medical Research Council for research projects to improve the physical health of people with a severe or persistent mental illness, providing funding of $AUD5.1 million over 5 years.
- Publication of a position statement by the Royal Australian College of General Practitioners on a Shared Care Models between GP and non-GP specialists for complex chronic conditions (47). It led to the RACGP/RANZCP Shared Care Model between GP and non-GP specialists for complex chronic conditions. Using new-onset schizophrenia as an exemplar it defines the roles, coordination and patient journey when GPs and specialists co-manage care.
- *Primary sense*, a software programme developed by Gold Coast PHN that ‘reads’ the GPs electronic computer record, analyses the record for patient safety issues and gaps in care. In real-time, GPs receive an alert or prompt to improve care. Outcomes show 47% action taken rate for alerts with the response rate maintained over at least 2 years. *Primary Sense* is installed in over 90% of Gold Coast PHN practises and is now being rolled out in 11 PHN areas (48). In a further system-wide step, Queensland Health is funding PHASES-Primary Sense project, establishing registries and registers to link cardiovascular screening, prevention, risk dashboard and outcome data system-wide across Queensland and potentially nationally.

Since the publication of the Roadmap, there has been a change in the Australian government and an emerging crisis in primary care service capability has become a national health priority. In this context, the take up of Roadmap policy proposals in the 2024 national budget reflects the extent to which they have had strong advocacy that has linked to the government’s agenda for reform.

Other developments indicate that sector stakeholders have also begun to implement recommendations through their own work and advocacy. The Roadmap recommendations provide a strong, consensus and evidence-based sector resource, including for the implementation of the Australian Government’s 5th National Mental Health and Suicide Prevention Plan and its successors. The systems-based, parity of esteem and consensus development approach used for the *Being Equally Well* project tackles complexity, fragmentation and skewed resourcing and provides stakeholders with evidence and tools with which to advocate for and to implement change.

Successfully implementing health policy at scale is increasingly understood to be dependent on the processes used to develop and implement it. The outcomes of the Being Equally Well Project suggest that the following three elements are critical to the design of health policy responses to complex and resistant health challenges that undermine both the health of individuals and the effectiveness of health care.

Firstly, a systems-based approach that recognises a system is, or interacting systems are, not only the sum of their own parts but the product of their interaction within each system and between them. This approach seeks to understand the processes, procedures, practises and policies that enable such complex relationships to be understood and improved.

Secondly, full engagement of those charged with delivering change, the frontline clinical staff. The quality and value of care produced by a large health system can be no better than the services generated by the small systems of which it is composed and particularly by those at the level of delivery of the desired improvements. Achievement of a seamless, patient-centred, high-quality, safe and efficient health care system, nested within the larger, disparate and fragmented but interacting service systems that provide governance and superstructure, requires the redesign and integration of disconnected components to provide a continuum of care to those who need it. Designing and implementing this requires the expertise and commitment of those who are intimately engaged in those processes.

Thirdly, equal input and trustworthy and productive interaction with patients, consumers and carers who will be the direct beneficiaries (49)Investment in an equitable, transparent, relational process involving all stakeholders as well as in the gathering of the best available evidence was a uniquely successful feature of the Being Equally Well approach (49). The experience of the project demonstrated that, when the experiences and different knowledge of professional and clinical experts and ‘experts by experience’ are recognised and valued, the application of evidence to a complex problem is considerably enhanced. The aspirations of consumers provided a core element to enable the selection of key priorities for improvement.

In conclusion, a codesign method that simultaneously integrated lived experience, frontline health provider expertise with health system leadership achieved a powerful consensus, enabling strong, authoritative leadership and advocacy for change.

## Data Availability

The original contributions presented in the study are included in the article/supplementary material, further inquiries can be directed to the corresponding author.
